# Phenolic Compounds Analysis and In Vitro Biological Activities of Aqueous Extract From Leaves of *Paronychia Arabica* Collected in South Algeria

**DOI:** 10.1002/fsn3.70912

**Published:** 2025-09-04

**Authors:** Walid Boussebaa, Zehour Rahmani, Saidi Mokhtar, Safia Ben Amor, Bachari Khaldoun, Abdellah Henni, Magda H. Abdellattif, Ayomide Victor Atoki, Wafa Zahnit, Mohammed Messaoudi

**Affiliations:** ^1^ Laboratory of Valorization and Promotion of Saharan Resources (VPRS), Faculty of Mathematics and Matter Sciences University of Ouargla Ouargla Algeria; ^2^ Scientific and Technical Research Center in Physico‐Chemical Analysis (CRAPC) Headquarters ex‐Pasna Industrial Zone Tipaza Algeria; ^3^ Higher School of Saharan Agriculture—El Oued El Oued Algeria; ^4^ Salama Lab Higher School of Saharan Agriculture—El Oued El Oued Algeria; ^5^ Laboratory of Dynamic Interactions and Reactivity of Systems University of Kasdi Merbah Ouargla Algeria; ^6^ Department of Chemistry, College of Science Taif University Taif Saudi Arabia; ^7^ Department of Biochemistry Kampala International University Ishaka Uganda; ^8^ Department of Chemistry, Faculty of Sciences University of Ferhat ABBAS Setif 1 Setif Algeria; ^9^ Chemistry Laboratory, Kouba Higher Normal School, Algiers Kouba Algeria

**Keywords:** α‐amylase enzyme activity, anti‐inflammotry impact, antimicrobial activity, antioxidant activity, LC–MS/MS analysis, *paronychia arabica*

## Abstract

The objective of this investigation was to assess the biological properties of the leaf's aqueous extract of *Paronychia Arabica* (PaAE), which is used in conventional medicine for therapeutic purposes of gastric ulcers and abdominal diseases. The content of phenolic and flavonoidic compounds was quantitatively estimated using colorimetric methods. The phenolic component profile was also evaluated using LC–MS/MS. The biological activities were then carried out, namely the antioxidant activity (DPPH test, β‐carotene bleaching and FRAP assays), the anti‐inflammatory impact, and the inhibitory effect of the α‐amylase enzyme was also examined. Lastly, antimicrobial activity against seven bacterial strains: *Pseudomonas aeruginosa* , *Escherichia coli* , *Klebsiella pneumoniae* , *Bacillus subtilis*, *Staphylococcus aureus* , *Listeria monocytogenes* , and *Micrococcus luteus* ; and one yeast: *Candida albicans* . The aqueous extract obtained by use of the decoction of leaves of *P. arabica* contains high levels of phenolics and flavonoids. Additionally, LC–MS–MS assessment of the phenolic profile revealed the existence of 16 chemical compounds and the major compounds are 35.9% cis‐p‐coumaric acid, 14.8% syringic acid, 9.61% sinapic acid, and 9.6% 8‐Hydroxyquinolin. The findings revealed that *P. arabica* extract has elevated antioxidant capacity in all tested samples; it also demonstrated a higher anti‐inflammatory activity. Antihyperglycemic activity is marked by a significant inhibitory effect on α‐amylase in vitro with an IC_50_ equal to 78.82 ± 3.4 μg/mL. Moreover, the *Staphylococcus aureus* strain had the highest sensitivity to *P. arabica* extract's antibacterial properties, whilst *Micrococcus luteus* and *Klebsiella pneumoniae* strains showed moderate sensitivity, and *Escherichia coli* displayed the greatest resistance.

## Graphical abstract

This study aimed to explore for the first time the in vivo and in vitro anti‐inflammatory, anti‐diabetic, and antimicrobial properties of Paronychia arabica. Positive correlation between the in vitro pharmacological activities that indicated the efficacy of *P. arabica* in α‐amylase inhibitory and denaturation of BSA. The phytochemical analysis revealed the simultaneous presence of Sinapic Acid and p‐coumaric acid in *P. arabica*. *P. arabica* extract is a promising alternative antidiabetic, antimicrobial, and anti‐inflammatory agent for the pharmaceutical industry.

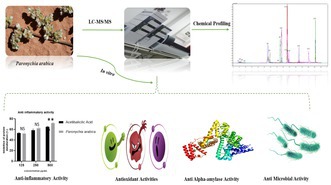

## 1 Introduction

To minimize the use of chemicals due to their undesirable effects, scientific progress is directed toward the use of natural products such as animal products like honey (Ben Amor et al. 2022), microorganisms, marine organisms, and plants in many fields, such as perfumery, pharmacology, and food processing, and to treat illnesses (Yuan et al. 2016). Phytotherapy is a recent discipline based on the utilization of medicinal and aromatic plants, which are mainly used to prepare medicines for the management of diverse medical conditions, as well as in the cosmetics and food sectors (Chhetri et al. 2015; Bouyahya et al. 2017). The beneficial benefits of medicinal plants are related to the existence of phytochemicals, particularly in the kinds that possess antioxidant properties including phenolic and flavonoid compounds (Otitolaiye et al. 2023). Several studies demonstrated the effects of these compounds such as antioxidant and antimicrobial (Lafarga et al. 2019; Alirezalu et al. 2020), anti‐inflammatory (Toma et al. 2020; Biluca et al. 2020), antidiabetic (Gulcin et al. 2019), anticholinesterase, and anti‐cancer (Bouhafsoun et al. 2018) actions.

High‐performance liquid chromatography (HPLC) was employed to detect and identify these phenolic substances (Katalinic et al. 2013). Recently, another technique developed for the structural identification of low and high molecular weight polyphenols in food samples is liquid chromatography combined with mass spectrometry (LC–MS) or tandem mass spectrometry (LC‐ MS/MS) (Motilva et al. 2013; Lucci et al. 2017).

In Algeria, medicinal plants have been utilized for ages to cure a variety of illnesses. Despite only 3164 plant species, Algeria is one of the wealthiest Arab countries (Benarba 2016).

*Paronychia arabica* belongs to the family of Caryophyllaceae, which contains over 2600 species in more than 80 genera (Jakimiuk et al. 2022). This species is very disturbed in Algeria. Some studies have demonstrated that *P. arabica* could be employed to improve human health as an antidiabetic (Afifi et al. 2005; Karar et al. 2020). It also is used in the treatment of several diseases such as stomach ulcers, bladder infections, anorexia, and prostate diseases (Bouanani et al. 2010; Elshamy et al. 2021). However, from our point of view, no work has been done to investigate phenolic compounds by chromatographic analysis, and no studies on the antimicrobial or anti‐inflammatory effect of these species have been carried out.

The primary objective of this research is to determine and quantify the principal phenolic substances using LC–MS/MS and assess the impacts of the aqueous extract of *P. arabica* on antioxidant activity using various tests, anti‐inflammatory, α‐amylase enzyme inhibitory, and antimicrobial activities against pathogenic strains. This plant is highly prized in traditional medicine as an aromatic plant with medicinal qualities.

## 2 Materials and Methods

### 2.1 Preparation of Extracts

*P. arabica* leaves were harvested in the Ouargla region, south Algeria, and identified by Prof. AIDOUD Amor (Department of agronomy, Faculty of nature and life, University of Ouargla (Algeria)). The voucher specimen was deposited at the laboratory of Valorization and Promotion of Saharan Resources (VPRS) Kasdi Merbah University, with the 2017 Ouargla/Par.ar herbarium number. The leaves were rinsed many times with tap water and distilled water to eliminate any remaining dust. Subsequently, the plant was desiccated at ambient temperature and converted into a powder.

Plant extract prepared by soaking 40 g of powder in 800 mL of H_2_O at 70°C for around 120 min, after which it was kept at room temperature for the next 24 h. Whatman filter paper n°1 was employed to capture and filter the supernatant. The filtrate was subsequently desiccated in a rotary evaporator at a temperature of 40°C–45°C. The remaining infusion was frozen and lyophilized. After being evaporated, the extract was stored at 4°C for additional use.

### 2.2 Dosage of Total Polyphenols Content (TPC)

TPC of the aqueous extract of *P. arabica* was determined using the Folin–Ciocalteu technique, which was modified slightly (Otitolaiye et al. 2023). Basically, 200 μL of PaAE/gallic acid mixed with 1.5 mL of 10% FCR. The mixture was kept in the dark for 5 min. Then 1.5 mL of 5% Na_2_CO_3_ was added and properly stirred. This was re‐covered and stored in a dark location at ambient temperature for 2 h. Subsequently, the absorbance was assessed at 750 nm.

The linear regression equation of the gallic acid calibration curve (displayed) was employed to estimate TPC (5, 10, 25, 50, 75, 100, 150, and 200 μg/mL). The quantity of phenol in the extracts was measured using the milligram equivalent of gallic acid (mgGAE/g extract). The assay was carried out three times.

### 2.3 Dosage of Total Flavonoids Content (TFC)

The AlCl_3_ colourimetric method was used to quantify the flavonoid content in this experiment (Djeridane et al. 2006). Briefly, 2 mL of 2% AlCl3 solution diluted in MeOH was combined with 2 mL of PaAE and incubated at ambient temperature for 30 min. The absorbance was determined at 415 nm. H_2_O was employed as blank, and quercetin was employed as the reference in a variety of concentrations from 10 to 300 μg/mL. The quantity of flavonoid in the extracts was calculated using the milligram equivalent of quercetin (mg QE/g extract). The assay was performed in triplicate.

### 2.4 Analysis of Phenolic Compounds by LC–MS/MS

#### 2.4.1 Solid Phase Extraction (SPE)

10 mg of lyophilized PaAEwas dissolved in 10 mL ultra‐pure water and purified by SPE vacuum using Isolut C 18 1 mg/3 mL SPE cartridge (previously conditioned). The retained polyphenols were eluted with 3 mL methanol grad LCMS and filtered with a nylon filter of 0.22 μm. The collected filtrate was injected into the LCMS/MS system.

#### 2.4.2 LC–MS/MS Instrumentation and Conditions

The UPLC‐ESI‐MS–MS Shimadzu 8040 (Shimadzu, Kyoto, Japan) was employed to conduct chromatographic examination of phenolic substances; Binary bump LC‐20 and Nexera XR was implemented in conjunction with ultra‐high sensitivity UFMS technology (Ultra‐Fast Mass Spectrometry). The ESI (Electro‐Spray Ionization) conditions were as follows:

- Nebulizing gas flow, 3.00 L/min
- CID gas, 230 KPs; conversion dynode
- Heat block, 400°C
−6.00 Kv; DL temperature, 250°C
- Drying gas flow, 15.00 L/min.

The MRM (Multiple Reaction Monitoring) mode was employed in the Ion mass trap spectrometer for both (±) (Negative/Positive ions).

#### 2.4.3 Chromatographic Conditions

The analytical column used for the chromatographic separation was Restek Raptor biphenyl 2.1 ID × 100 mm, 2.7 μm particle size.

35°C was the temperature of the oven. We used as mobile phase A (H_2_O, 5 mM NH_4_HCO_2_, and 0.1% CH_2_O_2_) as well as mobile phase B (MeOH, 5 mM NH_4_HCO_2_, and 0.1% CH_2_O_2_). The system gradient used is as follows: 0–2 min A 95%, 2–15 min A 5%, 15–18 min A 5%, 18–20 min A 95%. The flow rate was 0.4 mL/min.

### 2.5 In Vitro Pharmacological Properties

#### 2.5.1 Antioxidant Capacity

##### 2.5.1.1 DPPH Test

The DPPH test was performed to assess free radical scavenging activity, using the identical protocols as previously reported by Gali and Bedjou (Gali and Bedjou 2019), with some modifications.

Briefly, 1 mL of various concentrations of the PaAE (100–10 μg/mL) was added to the 1 mL of DPPH solution. After homogenization, the mixture was incubated in the dark for 30 min. The absorbance was then measured at 517 nm against a blank (distilled water). The IC_50_ values were used to determine the concentration of PaAE that scavenged 50% of the DPPH free radical, and this concentration was compared to that of the ascorbic acid and BHA (positive control).

The data was presented as a percentage of radical DPPH inhibition, which was calculated using the equation below:

$$\begin{array}{c} \text{Percentage of Inhibition}\%\\ \;=[\left(\text{control absorbance}-\text{extract absorbance}\right)/\\ \;\text{control absorbance}]\times 100 \end{array}$$

##### 2.5.1.2 Reducing Power Assay

The activity was determined using the method of Said et *al*. (Said et al. 2016). One mL of PaAE at several concentrations was combined with an equal volume of phosphate buffer (pH 6.6, 0.2 M) and 1% of K_3_Fe(CN)_6_. Following a 20‐min incubation at 50°C, 1 mL of 10% TCA was introduced, and the mixture was centrifuged at 3000 rpm for 10 min. A volume of 1.5 mL of H_2_O and 150 μL of 0.1% FeCl_3_ was introduced to the supernatant, and the absorbance was assessed at 700 nm, subsequently compared to that of the ascorbic acid (positive control). The concentration of the substrate that results in an absorbance of 0.5 at 700 nm is considered the reductive capacity (RC0.5). A linear regression curve is used to get it (Sait et al. 2015).

##### 2.5.1.3 β‐Carotene Bleaching Method

Linoleic acid oxidation generates radicals that can interact with β‐carotene, changing color from yellow to colorless. Antioxidants, however, inhibit the rate of β‐carotene degradation. This test was conducted according to the methodology described by Assaggaf et al. (2022).

A prepared solution of β‐carotene and linoleic acid was generated by dissolving 0.5 mg of β‐carotene in 25 μL of linoleic acid and 200 μL of Tween‐80 in 1 mL of chloroform. The solution was evaporated entirely under vacuum using a rotary evaporator at 40°C. Afterward, 100 mL of H_2_O was introduced to the solution mixture, which was then agitated.

A volume of 250 μL of PaAE at 1 mg/mL was added to test tubes, and a 5 mL aliquot of this mixture of reactions was put into the tubes. After a 2‐h incubation at 50°C, the absorbance of each sample was assessed at 470 nm relative to a control sample. In this examination, the standard utilized was butylated hydroxyanisole (BHA) alongside ascorbic acid. Antioxidant was expressed as percentage inhibition of β‐carotene degradation using the following formula:

$$\%\text{inhibition}=\left[\;A_{0}-A_{t}\right]/\left[{\mathrm{Ac}}_{0}-A_{\mathrm{ct}}\right]\times 100$$

%: inhibition.

*A*_0_: Absorbance of sample at T _0 min_.

*A*_t_: Absorbance of sample at T _60 min_.

Ac_0_: Absorbance of control at T _0 min_.

*A*_ct_: Absorbance of control at T _60 min_.

#### 2.5.2 Inhibition of Albumin Denaturation Activity

The inhibition of albumin denaturation approach was employed to investigate the anti‐inflammatory properties of PaAE, with slight alterations to the method of Chandra et al. (2012).

0.4 mL of fresh hen's egg albumin was combined with 0.8 mL of phosphate buffer (pH 6.4, 0.1 M) and 2 mL of PaAE at various concentrations, as well as 2 mL of H_2_O (Control). After 15 min of incubation at 37°C, the mixture was immediately immersed in a water bath at 70°C for 5 min. Subsequent to chilling, it was subjected to centrifugation for 10 min at 3000 rpm. The absorbance of the supernatant was assessed at 660 nm; as standard we used acetylsalicylic acid.

The percentage inhibition of protein denaturation was given as IC_50_ values (concentration necessary for 50% inhibition):

$$\begin{array}{c} \%=[\text{absorbance of control}—\text{absorbance of control}/\\ \;\text{absorbance of control}]\times 100 \end{array}$$

#### 2.5.3 Inhibition of α‐Amylase Activity

The α ‐amylase activity of an aqueous extract was determined using the method of Zengin et al. (Zengin et al. 2014) with minor modification. A volume of 25 μL of extract was added to 50 μL of α‐amylase solution (1 U/mL) in phosphate buffer (pH 6.9 with 6 mM sodium chloride). After pre‐incubation at 37°C for 10 min, the reaction was initiated with the addition of 50 μL of starch solution (0.1%) then, the mixture was incubated for the second time at 37°C for 10 min. A blank was prepared by combining PaAE with all the reagents of the process, omitting the enzyme (α‐amylase) solution. The process was then halted by the addition of 100 μL of IKI solution (iodine‐potassium iodide) and 25 μL of HCl (1 M). Acarbose was used as a reference inhibitor; the absorbance was read at 630 nm.

#### 2.5.4 Antibacterial Activity

The antimicrobial activity of *Pronychia arabica* extract was investigated against seven different types of pathogenic bacteria: *Escherichia coli* ATCC25922, *Pseudomonas aeruginosa* ATCC27853, and *Klebsiella pneumoniae* CIP8291 (Gram‐negative), *Staphylococcus aureus* ATCC43300, *Micrococcus luteus* ATCC 9314, *Bacillus subtilis* ATCC 6633, *Listeria monocytogenes* ATCC 13932 (Gram‐positive), and one yeast, *Candida albicans* ATCC10237.

The study was conducted using the disc diffusion procedure, which involves immersing discs in each sample. A pure and juvenile culture that is 18 h old was utilized to produce bacterial suspensions with an optical density of 0.5 McFarland (Baydar et al. 2004). A 1 mL volume of each microbial suspension was seeded on the surface of Muller–Hinton medium made of agar, prepared at a thickness of 4 mm, followed by a drying period of 3–5 min at ambient temperature. Subsequently, 3 disks (6 mm) saturated with the identical PaAE were positioned on the surface of the medium used for growth in the Petri dishes to ensure complete contact with the agar. A negative control was established by utilizing distilled water. Subsequently, Petri dishes were incubated at 37°C for 24 h after being pre‐distributed at 4°C for 3 h. The diameter of the inhibitory zone was measured in millimeters via a caliper.

### 2.6 Statistical Analyses

Results are presented as mean values ± SD from three measurements. The IC_50_ values were determined through linear regression analysis, and variance analyses were conducted using ANOVA with XLSTAT. Differences between means were assessed using the Tukey test, with *p* < 0.05 considered significant.

## 3 Results and Discussion

### 3.1 Total Phenolic and Flavonoid Contents

Plants produce phenolic substances, which may be found in large quantities in practically all plant parts. They are extensively used as food, as antibiotics, and for microbial diseases. These hydroxylated phenolic compounds have several benefits, including antioxidant, antiviral, and antimutagenic properties (Bursal et al. 2019). According to a quantitative study, the aqueous extract of *P. arabica* contains high levels of phenolic and flavonoid content, with 149.14 μg GAE/mg extract and 64.08 μg QE/mg extract, respectively.

A values of 525.80 ± 0.79 μg GAE/mg extract and 194.19 ± 8.62 μg QE/mg of methanolic extract of *Paronychia argentea* were recorded in phenolic and flavonoid contents, respectively, which were greater than those found in our findings (Adjadj et al. 2016).

Comparing these results with those of the various plant species of the same family, moreover, total phenolic contents of our plant are much higher than that indicated in the methanolic and acetonic extract of *Paronychia chionaea* (Karafakioglu et al. 2018). But we found that the phenolic content of our plant is lower than the phenolic content of *Paronychia mughlaei* (11.90 ± 0.3 and 7.39 ± 0.1 mg GAE/g extract) in the methanolic and aqueous extracts, respectively (Albayrak and Aksoy 2010); and for flavonoids, our content is higher compared with *Paronychia argentea* L (Adjadj et al. 2016).

The interpretation of this difference is due to climate and especially the areas of harvest. The two species, *Paronychia mughlaei* and *Paronychia chionaea*, are at the origin of Turkey (cold climate) on the other hand, our plant is harvested in the south of Algeria (arid zone). Another factor that causes this difference is the extraction method for the *Paronychia mughlaei* plant. The extraction is by Soxhlet for 6 h, which leads to higher yield and phenolic content.

Plant secretions are the primary source of phenolic compounds, which are classified as secondary metabolites (Ben Amor et al. 2022; Arkoub‐Hamitouche et al. 2020); the variation in total phenolic and flavonoid levels may be attributed to the location of the many floral sources, the timing of harvest, and the specific plant organ exploited (Yakoubi et al. 2021; Zengin et al. 2018).

### 3.2 Phenolic Compounds Profile

**FIGURE 1:**
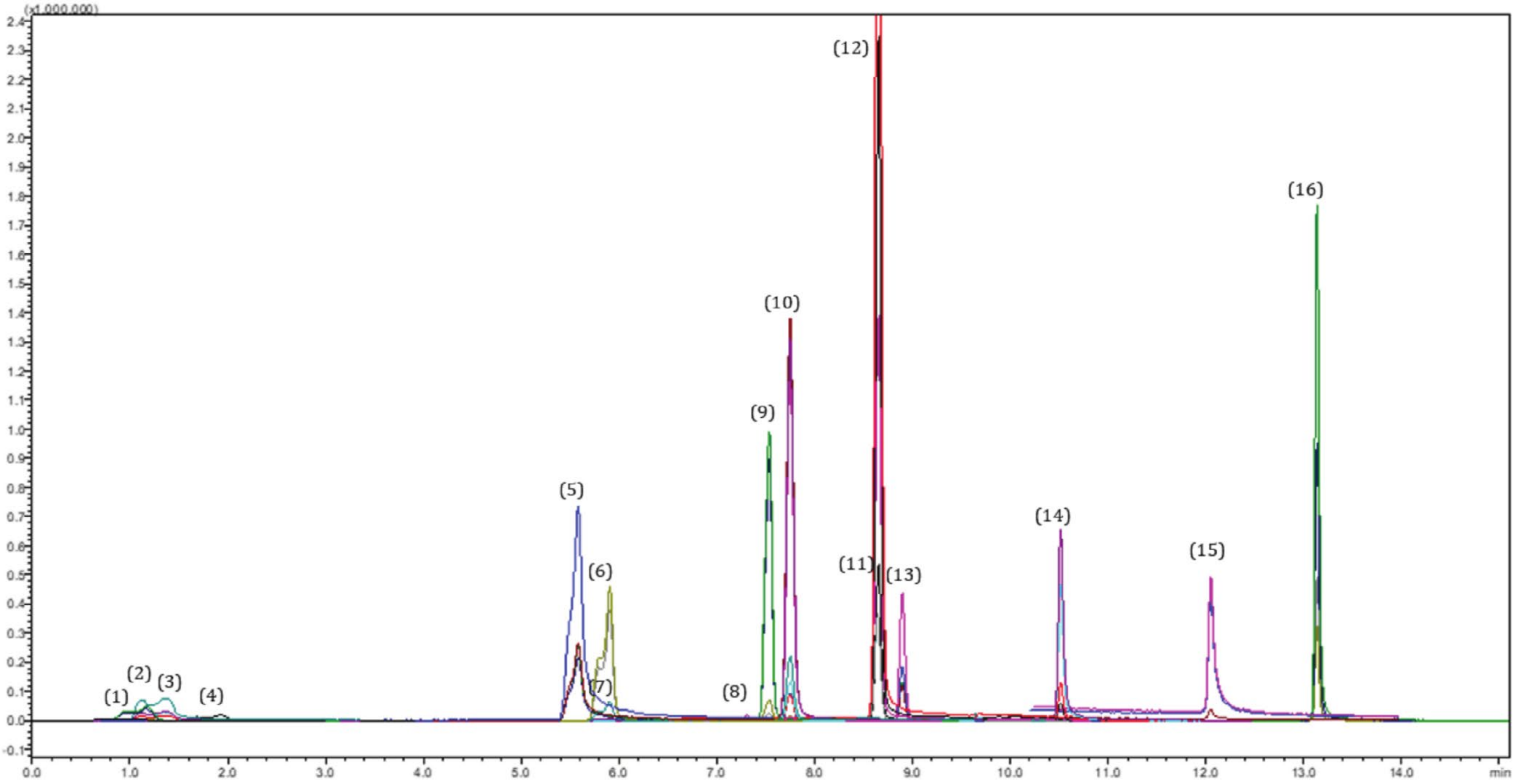
Total ion chromatogram (TIC) of 100 ppb standard. (1) Thymol, (2) Gallic acid, (3) Kojic acid, (4) 3,5‐Dihydroxybenzoic acid, (5) 8‐Hydroxyquinolin, (6) Caffeic acid, (7) Vanillic acid, (8) Syringic acid, (9) *Cis‐p‐coumaric* acid, (10) Naringenin, (11) Rutin, (12) 2‐Methoxybenzoic acid, (13) Sinapic acid, (14) Luteolin, (15) Salicylic acid, (16) Chrysin.

During this analysis, sixteen phenolic substance standards were analyzed and determined using LC–MS/MS (Figure 1). Retention times, parent ions, and fragment ions of the standards all affected identification peaks (Table 1).

**TABLE 1:** Detected phenolic compounds in PaAE using LC–MS/MS method.

Name	ESI Charge (±)	R.T (min)	Precursor ion > production	CE (v)	EQ	*R* ^2^	LOD/LOQ	RSD%
8‐Hydroxyquinolin	(+)	5.583	146.05 > 101.00	−33	*f*(*y*) = 223249 × *x−*44566.7	0.9989	0.15/0.46	2.8
Th*y*mol	(+)	0.976	151.75 > 74.2	−12	*f*(*y*) = 5158.86 × *x* + 16818.8	0.996	4.36/13.2	23.67
2‐Metho*x*ybenzoic Acid	(+)	8.625	153.05 > 135.05	−14	*f*(*y*) = 731512 × *x* + 434,458	0.9997	0.03/0.1	10.87
Kojic Acid	(+)	1.348	143.00 > 69.05	−18	*f*(*y*) = 34803.5 × *x−*26543.0	0,9923	1.62/4.9	7.98
Naringenin	(+)	7.751	272.95 > 177.00	−17	*f*(*y*) = 52896.5 × *x* + 57244.3	0.9954	0.14/0.43	15.42
Vanillic Acid	(−)	6.337	167.15 > 152.05	17	*f*(*y*) = 2361.67 × *x* + 1581.31	0,9959	1.79/5.43	11.26
Chrysin	(+)	13.135	255.05 > 153.05	−31	*f*(*y*) = 214484 × *x* + 123,895	0.9993	0.02/0.07	6.918
Rutin	(+)	8.651	611.20 > 73.20	−42	*f*(*y*) = 492.003 × *x−*795.623	0.997	1.11/3.36	18.19
Sinapic Acid	(−)	8.896	223.00 > 208.15	14	*f*(*y*) = 66773.2 × *x−*318.35	0.9987	0.01/0.04	4.37
3,5‐Dihydroxybenzoic Acid	(−)	1.934	153.10 > 109.10	15	*f*(*y*) = 17348.1 × *x−*7626.00	0.995	0.58/1.76	10.91
Caffiec Acid	(−)	5.906	179.15 > 135.05	17	*f*(*y*) = 200013 × *x−*4332.97	0.9976	0.08/0.24	3.27
Cis‐p. coumaric Acid	(−)	7.535	163.15 > 119.15	16	*f*(*y*) = 400237 × *x* + 26417.4	0.9986	0.02/0.07	2.07
Syringic Acid	(−)	7.313	196.95 > 182.00	14	*f*(*y*) = 7608.05 × *x* + 325.591	0.9899	0.22/0.66	18,19
Salysilic Acid	(−)	12.193	137.10 > 93.15	16	*f*(*y*) = 487918 × *x* + 37578.3	0.997	0.04/0.12	6.14
Gallic Acid	(−)	1.157	169.10 > 125.05	17	*f*(*y*) = 29990.4 × *x* + 18818.4	0.9998	1.42/4.29	12.4
Luteolin	(−)	10.510	284.95 > 133.00	36	*f*(*y*) = 159037 × *x−*12839.4	0.9967	0.04/0.12	2.58

Abbreviations: CE, collision energy; Eq, Equation; LDD/LDQ (μg/L), limit of detection/limit of quantification; *R* ^2^, coefficient of determination; RSDI, relative standard deviation; RT, retention time.

**FIGURE 2:**
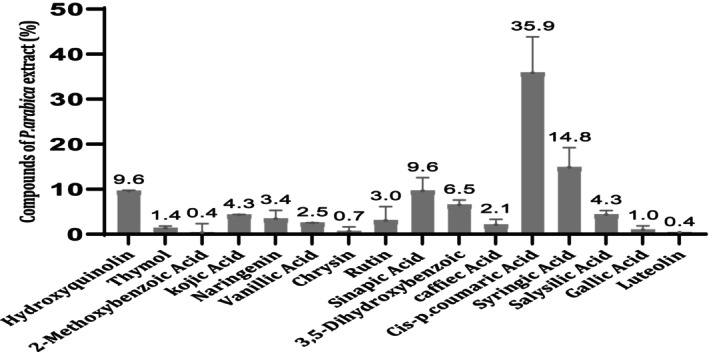
Percentage of phenolic compounds found in *Paronychia arabica* aqueous extract.

According to the results shown in Table 2, two categories of phenolic compounds were identified: phenolic acids and flavonoids. Phenolic acids represented a significant proportion of the polyphenolic compounds identified in PaAE, with 11 compounds including 8‐Hydroxyquinolin, 2‐Methoxybenzoic Acid, kojic Acid, Vanillic Acid, Sinapic Acid, 3,5‐Dihydroxybenzoic Acid, caffeic Acid, Cis‐p‐coumaric Acid, Syringic Acid, Salicylic Acid, and Gallic Acid, which represent more than 85% of the total compounds identified. Five compounds with a low percentage: Thymol, Naringenin, Chrysin, Rutin, and Luteolin (Figure 2) represented the flavonoid compounds.

**TABLE 2:** Quantification of phenolic compounds of *Paronychia arabica* aqueous extract (μg/mg) (Türkan et al. 2020).

ID	Name	Quantity (μg/mg)
1	8‐Hydroxyquinolin	31.23 ± 1.15
2	Thymol	4.453 ± 0.15
3	2‐Methoxybenzoic Acid	1.183 ± 0.46
4	Kojic Acid	13.993 ± 2.01
5	Naringenin	11.153 ± 0.10
6	Vanillic Acid	8.153 ± 1.88
7	Chrysin	2.153 ± 0.06
8	Rutin	9.883 ± 0.98
9	Sinapic Acid	31.253 ± 3.10
10	3,5‐Dihydroxybenzoic Acid	21.23 ± 2.95
11	Caffiec Acid	6.893 ± 1.04
12	Cis‐p. coumaric Acid	116.383 ± 11.2
13	Syringic Acid	48.163 ± 11.02
14	Salysilic Acid	14.103 ± 4.18
15	Gallic Acid	3.153 ± 0,91
16	Luteolin	1.243 ± 0,88

Among the sixteen detected compounds, the dominant among them was Cis‐p‐coumaric Acid with 116.383 ± 11.2 μg/mg (35.9% of total compounds). Next to *Cis‐p‐coumaric* acid, another dominant compound was Syringic acid (48.163 ± 11.02 μg/mg, 14.8%), followed by Sinapic acid with 31.253 ± 3.10 μg/mg and 8‐Hydroxyquinolin 31.23 ± 1.15 μg/mg. Among the flavonoids detected, Rutin is the most dominant with a content of 9.883 ± 0.98 μg/mg. The lowest level was detected in the 2‐Methoxybenzoic acid compound with 1.183 ± 0.46 μg/mg (Table 2 and Figure 2). Several studies have confirmed that botanical species belonging to the Caryophyllaceae family are very rich in phenolic compounds such as *p*‐coumaric acid and rutin (Jakimiuk et al. 2022; Benabderrahim et al. 2019).

Numerous studies have identified p‐coumaric acid as a significant phenolic component present in *Cinnamomum verum* (Gulcin et al. 2019); in *Chamaerops humilis* L (Bouhafsoun et al. 2018) and in an assemblage of therapeutic flora from arid and Saharan zones in Tunisia (Benabderrahim et al. 2019).

### 3.3 Biological Activities

#### 3.3.1 Antioxidant Activity

Due to its positive effects on human health and potential as a significant substitute for medications, which often have adverse effects, compounds derived from plants have garnered significant attention in recent years (Zengin et al. 2018; Magharbeh et al. 2020).

Many studies have employed the DPPH approach extensively to demonstrate the radical scavenging capacities of various substances. The degree of radical scavenging demonstrates the sample's antioxidant capability, which delays the start of the oxidation chain (Bursal et al. 2019). The extracts of *P. arabica* plants were compared with those of the ascorbic acid and BHA standards. The *P. arabica* aqueous extract had a remarkable capacity to scavenge the radical DPPH, with an IC_50_ equal to 20.92 ± 0.13 μg/mL, which exhibits no significant variation (*p* > 0.05) from the data found by the BHA (19.84 ± 0.21 μg/mL), but significantly (*p* < 0.05) higher than with ascorbic acid (4.67 ± 0.09 μg/mL) (Table 3). Ait Sidi Brahim et al. found a similar antioxidant activity of *Paronychia argentea* collected in the Moroccan region that equals 19.08 ± 0.62 μg/mL, which was greatest than Algerian *Paronychia argentea*, with values ranging from 27.38 to 144.92 μg/mL (Sait et al. 2015) and than Jordanian *Paronychia argentea* from 34.22 to 465.93 mg/mL (Magharbeh et al. 2020).

**TABLE 3:** Antioxidant activities of *Paronychia arabica* extract.

	*P. arabica*	BHA	Ascorbic Acid
DPPH (IC_50_μg/mL)	20.92 ± 0.13*,**	19.84 ± 0.21*,**	4.67 ± 0.09*
Reducing power assay (A0.5 μg/mL)	170 ± 0.56*,**	146 ± 0.89*,**	264 ± 1.07*
β‐carotene bleaching (IC_50_μg/mL)	282 ± 0.84*,**	2.911 ± 0.012*,**	64.04 ± 1.04*

*Note:* Data represent mean ± SEM (*n* = 3).

* *p* < 0.002 compared with control group.

** *p* < 0.05 compared with Ascorbic acid (Multiple comparison test followed by one‐way ANOVA).

**FIGURE 3:**
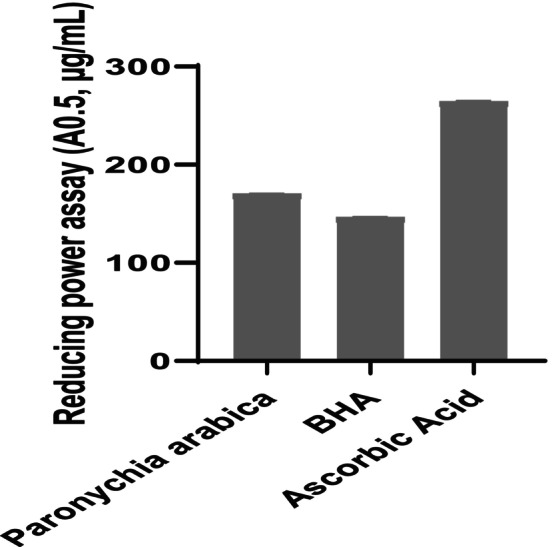
Reducing power of *Paronychia arabica* in comparison with BHA and ascorbic acid.

The reducing power of *P. arabica* extract and standards is shown in Figure 3 and Table 3. Aqueous extract and BHA had a high reducing power (RC0.5 = 170 ± 0.56 and 146 ± 0.89 μg/mL, respectively) compared to ascorbic acid with RC0.5 = 264 ± 1.07 μg/mL. Whereas the average RC0.5 of the *P. argentea* extracts levels ranged from 178 to 357 μg/mL (Sait et al. 2015).

**FIGURE 4:**
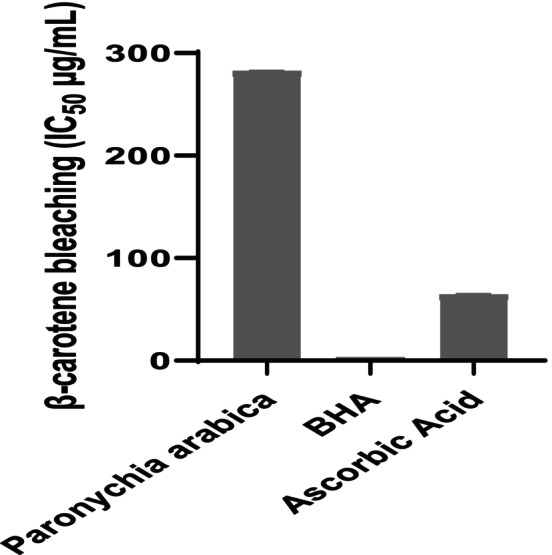
β‐Carotene bleaching test of *Paronychia arabica* in comparison with BHA and ascorbic acid.

In the presence of linoleic acid, the PaAE and BHA were able to obstruct the oxidation of β‐carotene in the β‐carotene bleaching test (Figure 4). The IC_50_ of the aqueous extract (282 ± 0.84 μg/mL) was higher than that of the standard BHA (2.911 ± 0.012 μg/mL) and ascorbic acid (64.04 ± 1.04 μg/mL). Numerous studies have demonstrated that BHA exhibits significant inhibitory ability toward β‐carotene bleaching (Otitolaiye et al. 2023; Zengin et al. 2018).

The β ‐carotene–linoleic acid system is reported for *P. arabica* for the first time. The rapid degradation of beta‐carotene results from the lack of antioxidants. Our results suggested that the IC_50_ values of the extract demonstrated a moderate protective effect on β‐carotene compared to the standards (BHA and Ascorbic acid). This may be attributed to several antioxidants: those that neutralize peroxyl radicals, produce free radicals of linoleate, and other chemicals inside the system. The current investigation demonstrated that PaAE had a substantial amount of phenolic compounds that inhibited lipoperoxidation and, consequently, the oxidation of β ‐carotene.

The Paronychia genus is known for its secondary metabolites, such as phenolic acids and flavonoids, which display various antioxidant properties. It is important to highlight that electron‐donating groups, including hydroxyl (OH), methoxy (OCH_3_), and alkyl groups, significantly lower the redox potential of phenolic compounds, thereby improving their antioxidant properties (Akintola et al. 2020). Consequently, it is essential to acknowledge the direct correlation between the concentration of phenolic compounds and the antioxidant capacity. As previously articulated in the results of the LC–MS/MS and HPLC‐UV analyses, the predominant chemical constituents identified in the PaAE are flavonoids and phenolic acids.

The sources of natural antioxidants, including phytochemicals like flavonoids, phenolics, and anthocyanins, have attracted a lot of attention. It has been observed that plant materials such as green vegetables, fruits, seeds, and grains contain natural antioxidants (Akintola et al. 2020; Gheraissa et al. 2023).

Another reason attributes to the presence of naringenin in *P. arabica* extract; it promotes antioxidant defense function of enzymes and elevates glutathione levels, thereby mitigating STZ‐induced liver complications (Rashmi et al. 2018).

On the other hand, numerous flavonoids identified in PaAE using LC–MS/MS, including Rutin, Luteolin, and 2‐Methoxybenzoic Acid, undergo significant degradation into different phenolic acids, some of which retain radical‐scavenging properties. Both the absorbed flavonoids and their metabolites may exhibit in vitro antioxidant action (Van Acker et al. 1996; Zahnit et al. 2023).

#### 3.3.2 Inhibition of Albumin Denaturation Activity

**FIGURE 5:**
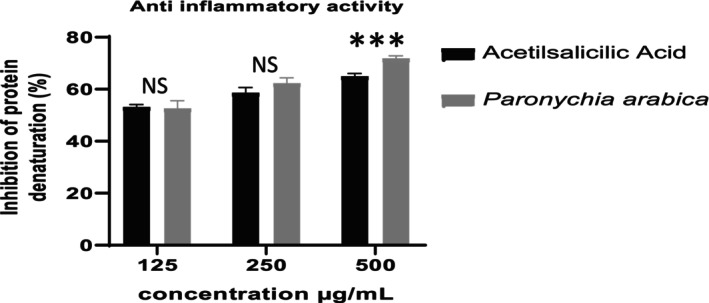
Inhibition of albumin denaturation activity of *Paronychia arabica*. NS: not signifcant; ***: highly significant.

The anti‐inflammatory action of PaAE was tested in vitro against the denaturation of egg albumin in this study. The results of anti‐inflammatory ratios for a *P. arabica* aqueous extract and Acid Acetilsalicilic at concentrations of 125, 250, and 500 g/mL show that the anti‐inflammatory activity increases with increasing concentrations, with ratios of 52.47%, 62.19%, and 71.69% for the extract, and acid acetilsalicilic has ratios of 53.01%, 58.53%, and 64.81% (Figure 5).

This was validated further by comparing their IC_50_ values. *P. arabica* extract had an IC_50_ value of 24 μg/mL, whereas acetilsalicilic acid had a value of 101 μg/mL. As a result, it had the greatest impact because it outperformed the usual reference. Chandra et al. (2012) found similar findings whose IC_50_ of *Coffea arabica* extract is lower than those of the standard.

Key compounds of PaAE, such as gallic acid, luteolin, naringenin, and synapic acid, have been identified in the treatment of asthma through the inhibition of various anti‐inflammatory targets, including SELE, IL‐2, and CXCL10, at both mRNA and protein levels. These compounds play a role in biological processes related to immune response, inflammatory response, cell–cell signaling, and reaction to lipopolysaccharide.

The advantageous impacts of chrysin, which is the major identified component in PaAE, play a key function in the inhibition of levels in the blood and are noteworthy. The nuclear transcription factor κB (NF‐κB) p65 unit, tumor necrosis factor alpha (TNF‐α), interleukin‐1β (IL‐1β), IL‐6, IL‐12, IL‐17A, and interferon gamma (IFN‐γ) have been revealed (Zeinali et al. 2017).

The extracts' capacity to inhibit protein denaturation is related to their chemical composition, which works as a protein stabilizer. Because albumin is alkaline, the nature of the bondable chemicals, such as phenolic acids (Perna et al. 2012; Tatlow et al. 2015). This agrees well with our qualitative and quantitative LC–MS/MS phenolic compounds result, which is very rich in phenolic acids (Table 2).

#### 3.3.3 Inhibition of α‐Amylase Activity

The hypoglycemic effect of medicinal plants may be attributed to reduced sugar absorption. This latter may be accomplished by inhibiting enzymes that breakdown complex polysaccharides. Since a long time, one of these enzymes, alpha amylase, has been taken into consideration as a possible target for the management of diabetes (Van Acker et al. 1996; Zahnit et al. 2023).

PaAE exhibits a pronounced inhibitory action on the activity of the α‐amylase enzyme, demonstrating an IC_50_ level of 78.82 ± 3.4 and differing statistically from acarbose 228.07 ± 0.89 μg/mL. This inhibitory effect is comparable to acarbose, a blood glucose‐reducing medication used in diabetic patients.

The hypoglycaemic effect of *Paronychia argentea* was mediated by inhibition of α‐amylase activity; however, further research in vivo (Hamdan and Afifi 2004) and in vitro proved this to be false (Veeraraghavan et al. 2020).

According to several studies, the capacity of *Paronychia argentea* was ascribed to its elevated flavonoid content; especially isorhamnetin, quercetin, and luteolin (Veeraraghavan et al. 2020; Abu Soud et al. 2004). The results of Kim et al. (Kim et al. 2000) demonstrated that 0.5 mg/mL luteolin suppressed 36% of α‐amylase activity, which was less than acarbose's impact.

Our result is superior to that found in the extract from *Heracleum persicum* and *Ziziphus jujuba* with IC_50_ values of 307 and 867 μg/mL respectively (Afrisham et al. 2015) and differs from the results of *Artemisia campestris* L collected in the Saharan Algeria region ranging from 11.79 ± 0.14 to 284.33 ± 3.9 μg/mL (Zahnit et al. 2022).

#### 3.3.4 Antibacterial Activity

Given the escalating antibiotic resistance of pathogenic microorganisms and the scarcity of therapeutic alternatives, novel treatment tactics are imperative (Ben Amor et al. 2022; Al‐Masaudi et al. 2020; Sharaf et al. 2021). Medicinal plants contain a wide range of chemical components that have been shown in vitro to have antibacterial properties (Lewis and Ausubel 2006; Almabruk et al. 2018; Vaou et al. 2021).

**FIGURE 6:**
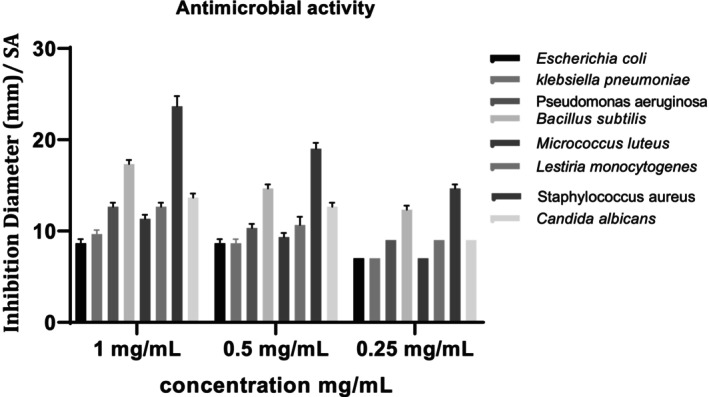
Antibacterial activity of *Paronychia arabica* extract against microbial strains studied. *p*‐Coumaric acid and its derivatives exhibit several bioactive features, including antioxidant, antibacterial, and anti‐inflammatory effects. Due to its significant bioactive potential, p‐coumaric acid (the primary bioactive component in PaAE) could be responsible for the pharmacological actions seen in *P. arabica* extract (Kaur and Kaur 2022).

The results of this study found that *P. arabica* extract has antimicrobial effects on both the Gram‐negative and Gram‐positive bacteria and yeast examined, but meilleur inhibition was noted in Gram‐positive ones (Figure 6).

Among gram‐positive bacteria, *Staphylococcus aureus* was the most affected by *P. arabica* extract, with the most inhibitory zone impact measured from 23 ± 1.11 to 14.66 ± 0.44 mm. This bacterium was inhibited by different kinds of honey (Ben Amor et al. 2022), by *Cinnamomum tamala* extract (Manandhar et al. 2019) and some medicinal plants (Gheraissa et al. 2023). In addition, *B. subtilis* had a remarkable inhibition zone arriving at 17.33 ± 0.44 mm. *Micrococcus luteus* and *Listeria monocytogenes* were less sensitive to the other pathogenic gram‐positive bacteria, whereby the best inhibition diameter was noticed in *L. monocytogenes* (12.66 ± 0.44 mm) (Figure 6).

This finding is highly consistent with the literature, which shows that Gram‐positive bacteria are more responsive to fruit PC's antimicrobial properties than Gram‐negative bacteria (Wafa et al. 2017; Lima et al. 2019).

Low inhibition zones were recorded in gram‐negative bacteria ranging from 12.66 ± 0.44 to 7 ± 0.00 mm, including *Pseudomonas aeruginosa, which* was the most affected strain.

Numerous studies regarding showed that phenolic compounds, such as rutin, hydroxytyrosol, caffeic acid, and oleuropein have great antimicrobial activity against bacteria strains (Karygianni et al. 2014; Borjan et al. 2020).

*Candida albicans* is the most pathogenic species in the genus Candida, causing candidiasis in humans and animals (Rane et al. 2013). *Candida albicans* was moderately inhibited by our plant extract, with zones of inhibition varying between 13.66 ± 0.66 mm.

Several studies have demonstrated the effectiveness of plant extracts against this yeast (Jahani et al. 2017; Gizaw et al. 2022).

Diabetes is a metabolic disorder characterized by persistent hyperglycemia. There are several medical approaches for the treatment of type 2 diabetes. Inhibiting alpha‐amylase activity represents a singular approach to reducing postprandial blood glucose levels (Funke and Melzig 2006). We can use inhibitors of this enzyme to treat obesity and diabetes. It catalyzes the hydrolysis of α‐(1,4)‐D‐glycosidic linkages in starch and other glucose polymers in diabetic patients. The inhibition of alpha‐amylase prevents the breakdown of starch, which leads to a decrease in blood glucose levels (Mahmood 2016). A prior investigation has established the involvement of flavonoids in the inhibition of alpha‐amylase activity. It identified chrysin, a component of PaAE, as a potent flavonoid inhibitor of alpha‐amylase and explored the potential for its delivery in concentrated form (capsules with food intake) to mitigate postprandial hyperglycemia by inhibiting starch digestion. The proposed action mechanism for the inhibitory ability of flavonoids relates to the quantity of hydroxyl groups on the B ring of the flavonoid structure, the establishment of hydrogen bonds between the hydroxyl groups of the polyphenol ligands, and the catalytic residues present at the enzyme's binding site (Gu et al. 2015). Giving syringic acid, which is identified in PaAE (50 mg/kg b.w.), to diabetic rats by mouth for 30 days changes their blood sugar levels in a favorable way. These results show that syringic acid may be able to address problems with glycoprotein components in addition to lowering blood sugar levels in experimental diabetes (Sales et al. 2012). Compared to previous antidiabetic investigations on PaAE or other species, none of them used antidiabetic approaches to illustrate how effective and strongly bioactive the extract of interest is.

## 4 Conclusion

This paper is unique in that it provides the first quantitative and qualitative investigation of phenolic content using LC–MS/MS analysis and evaluates the broad spectrum of biological activities (in vitro) of *P. arabica* growing in south Algeria.

The results of this study justified that *P. arabica* extract revealed strong biological activities, notably anti‐inflammatory and anti‐diabetic action in the first instance, as well as antioxidant and antimicrobial activities. This could be due to the high levels of polyphenols and flavonoids.

In addition to its use in traditional medicine, we suggest that this plant's phytochemicals and biological activity will serve as a source of inspiration for drug developers who wish to treat conditions including diabetes mellitus, bacterial diseases, and dementia symptoms, and further research on the precise mechanisms of their in vivo therapeutic effects is required. Consequently, we suggest that the plant be kept and safeguarded, primarily because it benefits human health and the ecology.

## Author Contributions

**Walid Boussebaa:** conceptualization (equal), data curation (equal), writing – original draft (equal). **Zehour Rahmani:** conceptualization (equal), investigation (equal), methodology (equal). **Saidi Mokhtar:** formal analysis (equal). **Safia Ben Amor:** formal analysis (equal), software (equal). **Bachari Khaldoun:** formal analysis (equal). **Abdellah Henni:** conceptualization (equal), resources (equal). **Magda H. Abdellattif:** investigation (equal). **Ayomide Victor Atoki:** writing – review and editing (equal). **Wafa Zahnit:** formal analysis (equal).

## Conflicts of Interest

The authors declare no conflicts of interest.

## Data Availability

We confirm that all the data and findings of this study are available within the article.

## References

[fsn370912-bib-0001] Abu Soud, R. S. , L. I. Hamdan , and F. U. Afifi . 2004. “Alpha Amylase Inhibitory Activitv of Some Plant Extracts With Hyporrlycemic Activity.” Scientia Pharmaceutica 72, no. 1: 25–33.

[fsn370912-bib-0002] Adjadj, M. , A. Baghiani , S. Boumerfeg , et al. 2016. “Protective Effect of Paronychia Argentea L. on Acetic Acid Induced Ulcerative Colitis in Mice by Regulating Antioxidant Parameters and Inflammatory Markers.” Pharma Chemica 8, no. 4: 207–218.

[fsn370912-bib-0003] Afifi, F. U. , B. Al‐Khalidi , and E. Khalil . 2005. “Studies on the In Vivo Hypoglycemic Activities of Two Medicinal Plants Used in the Treatment of Diabetes in Jordanian Traditional Medicine Following Intranasal Administration.” Journal of Ethnopharmacology 100, no. 3: 314–318. 15885943 10.1016/j.jep.2005.03.016

[fsn370912-bib-0004] Afrisham, R. , M. Aberomand , M. A. Ghaffari , A. Siahpoosh , and M. Jamalan . 2015. “Inhibitory Effect of Heracleum Persicum and *Ziziphus Jujuba* on Activity of Alpha‐Amylase.” Journal of Botany 2015: 824683.

[fsn370912-bib-0005] Akintola, A. O. , B. D. Kehinde , P. B. Ayoola , et al. 2020. “Antioxidant Properties of Silver Nanoparticles Biosynthesized From Methanolic Leaf Extract of *Blighia Sapida* .” In IOP Conference Series: Materials Science and Engineering, 012004. IOP Publishing.

[fsn370912-bib-0007] Albayrak, S. , and A. Aksoy . 2010. “In Vitro Antioxidant and Antimicrobial Properties of Paronychia Mughlaei Chaudhri.” Acta Botanica Gallica 157, no. 3: 411–418.

[fsn370912-bib-0008] Alirezalu, A. , N. Ahmadi , P. Salehi , et al. 2020. “Physicochemical Characterization, Antioxidant Activity, and Phenolic Compounds of Hawthorn (Crataegus spp.) Fruits Species for Potential Use in Food Applications.” Food 9, no. 4: 436. 10.3390/foods9040436 PMC7230283 32260449

[fsn370912-bib-0009] Almabruk, K. H. , L. K. Dinh , and B. Philmus . 2018. “Self‐Resistance of Natural Product Producers: Past, Present, and Future Focusing on Self‐Resistant Protein Variants.” ACS Chemical Biology 13, no. 6: 1426–1437. 29763292 10.1021/acschembio.8b00173

[fsn370912-bib-0010] Al‐Masaudi, S. B. , M. B. Hussain , S. M. Al‐Maaqar , S. Al Jaouni , and S. Harakeh . 2020. “In Vitro Antibacterial Activity of Honey Against Multidrug‐Resistant *Shigella sonnei* .” Complementary Therapies in Clinical Practice 41: 101257. 10.1016/j.ctcp.2020.101257. 33157353

[fsn370912-bib-0011] Arkoub‐Hamitouche, L. , V. González‐del‐Campo , M. E. López‐Oliva , F. Bedjou , and O. M. Palomino . 2020. “Paronychia Argentea Lam. Protects Renal Endothelial Cells Against Oxidative Injury.” Journal of Ethnopharmacology 248: 112314. 31629861 10.1016/j.jep.2019.112314

[fsn370912-bib-0012] Assaggaf, H. M. , H. Naceiri Mrabti , B. S. Rajab , et al. 2022. “Singular and Combined Effects of Essential Oil and Honey of *Eucalyptus globulus* on Anti‐Inflammatory, Antioxidant, Dermatoprotective, and Antimicrobial Properties: In Vitro and In Vivo Findings.” Molecules 27, no. 16: 5121. 36014359 10.3390/molecules27165121 PMC9415335

[fsn370912-bib-0013] Baydar, N. G. , G. Özkan , and O. Sağdiç . 2004. “Total Phenolic Contents and Antibacterial Activities of Grape (*Vitis vinifera* L.) Extracts.” Food Control 15, no. 5: 335–339. 10.1016/S0956-7135(03)00083-5.

[fsn370912-bib-0014] Ben Amor, S. , S. Mekious , L. Allal Benfekih , et al. 2022. “Phytochemical Characterization and Bioactivity of Different Honey Samples Collected in the Pre‐Saharan Region in Algeria.” Life 12, no. 7: 927. 10.3390/life12070927. 35888017 PMC9321394

[fsn370912-bib-0015] Benabderrahim, M. A. , Y. Yahia , I. Bettaieb , W. Elfalleh , and K. Nagaz . 2019. “Antioxidant Activity and Phenolic Profile of a Collection of Medicinal Plants From Tunisian Arid and Saharan Regions.” Industrial Crops and Products 138: 111427.

[fsn370912-bib-0016] Benarba, B. 2016. “Medicinal Plants Used by Traditional Healers From South‐West Algeria: An Ethnobotanical Study.” Journal of Intercultural Ethnopharmacology 5, no. 4: 320–330. 27757260 10.5455/jice.20160814115725 PMC5061473

[fsn370912-bib-0017] Biluca, F. C. , B. da Silva , T. Caon , et al. 2020. “Investigation of Phenolic Compounds, Antioxidant and Anti‐Inflammatory Activities in Stingless Bee Honey (Meliponinae).” Food Research International 129: 108756. 32036884 10.1016/j.foodres.2019.108756

[fsn370912-bib-0018] Borjan, D. , M. Leitgeb , Ž. Knez , and M. K. Hrnčič . 2020. “Microbiological and Antioxidant Activity of Phenolic Compounds in Olive Leaf Extract.” Molecules 25, no. 24: 5946. 33334001 10.3390/molecules25245946 PMC7765412

[fsn370912-bib-0019] Bouanani, S. , C. Henchiri , E. Migianu‐Griffoni , N. Aouf , and M. Lecouvey . 2010. “Pharmacological and Toxicological Effects of Paronychia Argentea in Experimental Calcium Oxalate Nephrolithiasis in Rats.” Journal of Ethnopharmacology 129, no. 1: 38–45. 20138208 10.1016/j.jep.2010.01.056

[fsn370912-bib-0020] Bouhafsoun, A. , M. A. Yilmaz , A. Boukeloua , H. Temel , and M. K. Harche . 2018. “Simultaneous Quantification of Phenolic Acids and Flavonoids in *Chamaerops Humilis* L. Using LC–ESI‐MS/MS.” Food Science and Technology 38: 242–247.

[fsn370912-bib-0021] Bouyahya, A. , J. Abrini , A. Et‐Touys , Y. Bakri , and N. Dakka . 2017. “Indigenous Knowledge of the Use of Medicinal Plants in the North‐West of Morocco and Their Biological Activities.” European Journal of Integrative Medicine 13: 9–25.

[fsn370912-bib-0022] Bursal, E. , A. Aras , Ö. Kılıç , P. Taslimi , A. C. Gören , and İ. Gülçin . 2019. “Phytochemical Content, Antioxidant Activity, and Enzyme Inhibition Effect of Salvia Eriophora Boiss. & Kotschy Against Acetylcholinesterase, α‐Amylase, Butyrylcholinesterase, and α‐Glycosidase Enzymes.” Journal of Food Biochemistry 43, no. 3: e12776. 31353544 10.1111/jfbc.12776

[fsn370912-bib-0023] Chandra, S. , P. Chatterjee , P. Dey , and S. Bhattacharya . 2012. “Evaluation of In Vitro Anti‐Inflammatory Activity of Coffee Against the Denaturation of Protein.” Asian Pacific Journal of Tropical Biomedicine 2, no. 1: S178–S180.

[fsn370912-bib-0024] Chhetri, B. K. , N. A. Awadh Ali , and W. N. Setzer . 2015. “A Survey of Chemical Compositions and Biological Activities of Yemeni Aromatic Medicinal Plants.” Medicines (Basel) 2, no. 2: 67–92. 28930202 10.3390/medicines2020067 PMC5533162

[fsn370912-bib-0025] Djeridane, A. , M. Yousfi , B. Nadjemi , S. Maamri , F. Djireb , and P. Stocker . 2006. “Phenolic Extracts From Various Algerian Plants as Strong Inhibitors of Porcine Liver Carboxylesterase.” Journal of Enzyme Inhibition and Medicinal Chemistry 21, no. 6: 719–726. 10.1080/14756360600810399. 17252945

[fsn370912-bib-0026] Elshamy, A. I. , T. A. Mohamed , M. A. A. Ibrahim , et al. 2021. “Two Novel Oxetane Containing Lignans and a New Megastigmane From *Paronychia Arabica* and *in Silico* Analysis of Them as Prospective SARS‐CoV‐2 Inhibitors.” RSC Advances 11, no. 33: 20151–20163. 10.1039/D1RA02486H. 35479905 PMC9033657

[fsn370912-bib-0027] Funke, I. , and M. F. Melzig . 2006. “Traditionally Used Plants in Diabetes Therapy: Phytotherapeutics as Inhibitors of Alpha‐Amylase Activity.” Revista Brasileira de Farmacognosia 16: 1–5.

[fsn370912-bib-0028] Gali, L. , and F. Bedjou . 2019. “Antioxidant and Anticholinesterase Effects of the Ethanol Extract, Ethanol Extract Fractions and Total Alkaloids From the Cultivated *Ruta chalepensis* .” South African Journal of Botany 120: 163–169.

[fsn370912-bib-0029] Gheraissa, N. , A. E. Chemsa , N. Cherrada , et al. 2023. “Biochemical Profile and In Vitro Therapeutic Properties of Two Euhalophytes, *Halocnemum strobilaceum* Pall. And *Suaeda fruticosa* (L.) Forske., Grown in the Sabkha Ecosystem in the Algerian Sahara.” Molecules 28, no. 8: 3580. 37110814 10.3390/molecules28083580 PMC10141351

[fsn370912-bib-0030] Gizaw, A. , L. M. Marami , I. Teshome , et al. 2022. “Phytochemical Screening and In Vitro Antifungal Activity of Selected Medicinal Plants Against Candida Albicans and Aspergillus Niger in West Shewa Zone, Ethiopia.” Advances in Pharmacological and Pharmaceutial Sciences 2022: 1–8. 10.1155/2022/3299146 PMC9256430 35800399

[fsn370912-bib-0031] Gu, C. , H. Zhang , C. Y. Putri , and K. Ng . 2015. “Evaluation of α‐Amylase and α‐Glucosidase Inhibitory Activity of Flavonoids.” International Journal of Food and Nutritional Sciences 2: 1–6.

[fsn370912-bib-0032] Gulcin, I. , R. Kaya , A. C. Goren , et al. 2019. “Anticholinergic, Antidiabetic and Antioxidant Activities of Cinnamon ( *Cinnamomum verum* ) Bark Extracts: Polyphenol Contents Analysis by LC‐MS/MS.” International Journal of Food Properties 22, no. 1: 1511–1526.

[fsn370912-bib-0033] Hamdan, I. I. , and F. U. Afifi . 2004. “Studies on the In Vitro and In Vivo Hypoglycemic Activities of Some Medicinal Plants Used in Treatment of Diabetes in Jordanian Traditional Medicine.” Journal of Ethnopharmacology 93, no. 1: 117–121. 15182916 10.1016/j.jep.2004.03.033

[fsn370912-bib-0034] Jahani, S. , S. Bazi , Z. Shahi , M. S. Asadi , F. Mosavi , and G. S. Baigi . 2017. “Antifungal Effect of the Extract of the Plants Against *Candida albicans* .” International Journal of Infection 4, no. 2: e36807.

[fsn370912-bib-0035] Jakimiuk, K. , M. Wink , and M. Tomczyk . 2022. “Flavonoids of the Caryophyllaceae.” Phytochemistry Reviews 21, no. 1: 179–218.

[fsn370912-bib-0037] Karafakioglu, Y. S. , L. Aksoy , and M. Kargioglu . 2018. “Antioxidant Activity and Mineral Ingredient Assessment of Different Solvent Extracts of Paronychia Chionaea.” Pakistan Journal of Botany 50, no. 5: 1913–1916.

[fsn370912-bib-0038] Karar, A. , A. Henni , F. Namoune , and F. Rosei . 2020. “Inhibition of Nucleation and Crystal Growth of Calcium Carbonate in Hard Waters Using *Paronychia arabica* in an Arid Desert Region.” Water Environment Journal 34, no. S1: 979–987. 10.1111/wej.12596.

[fsn370912-bib-0039] Karygianni, L. , M. Cecere , A. L. Skaltsounis , et al. 2014. “High‐Level Antimicrobial Efficacy of Representative Mediterranean Natural Plant Extracts Against Oral Microorganisms.” BioMed Research International 2014: 1–8. 10.1155/2014/839019 PMC4098616 25054150

[fsn370912-bib-0040] Katalinic, V. , S. S. Mozina , I. Generalic , D. Skroza , I. Ljubenkov , and A. Klancnik . 2013. “Phenolic Profile, Antioxidant Capacity, and Antimicrobial Activity of Leaf Extracts From Six *Vitis vinifera* L. Varieties.” International Journal of Food Properties 16, no. 1: 45–60.

[fsn370912-bib-0041] Kaur, J. , and R. Kaur . 2022. “P‐Coumaric Acid: A Naturally Occurring Chemical With Potential Therapeutic Applications.” Current Organic Chemistry 26, no. 14: 1333–1349.

[fsn370912-bib-0042] Kim, J.‐S. , C.‐S. Kwon , and K. H. Son . 2000. “Inhibition of Alpha‐Glucosidase and Amylase by Luteolin, a Flavonoid.” Bioscience, Biotechnology, and Biochemistry 64, no. 11: 2458–2461. 11193416 10.1271/bbb.64.2458

[fsn370912-bib-0043] Lafarga, T. , S. Villaró , G. Bobo , J. Simó , and I. Aguiló‐Aguayo . 2019. “Bioaccessibility and Antioxidant Activity of Phenolic Compounds in Cooked Pulses.” International Journal of Food Science and Technology 54, no. 5: 1816–1823.

[fsn370912-bib-0044] Lewis, K. , and F. M. Ausubel . 2006. “Prospects for Plant‐Derived Antibacterials.” Nature Biotechnology 24, no. 12: 1504–1507. 10.1038/nbt1206-1504 17160050

[fsn370912-bib-0045] Lima, M. d. C. , C. P. de Sousa , C. Fernandez‐Prada , et al. 2019. “A Review of the Current Evidence of Fruit Phenolic Compounds as Potential Antimicrobials Against Pathogenic Bacteria.” Microbial Pathogenesis 130: 259–270. 30917922 10.1016/j.micpath.2019.03.025

[fsn370912-bib-0046] Lucci, P. , J. Saurina , and O. Núñez . 2017. “Trends in LC‐MS and LC‐HRMS Analysis and Characterization of Polyphenols in Food.” TrAC, Trends in Analytical Chemistry 88: 1–24. 10.1016/j.trac.2016.12.006.

[fsn370912-bib-0047] Magharbeh, M. K. , T. A. Al‐Hujran , S. M. Al‐Dalaen , and A.‐W. R. Hamad . 2020. “Assessment of Paronychia Argentea Extraction on Kidney Stone by Using Calcium Oxalate Method.” Biomedical Pharmacology Journal 13, no. 4: 1745–1754.

[fsn370912-bib-0048] Mahmood, N. 2016. “A Review of α‐Amylase Inhibitors on Weight Loss and Glycemic Control in Pathological State Such as Obesity and Diabetes.” Comparative Clinical Pathology 25, no. 6: 1253–1264.

[fsn370912-bib-0049] Manandhar, S. , S. Luitel , and R. K. Dahal . 2019. “In Vitro Antimicrobial Activity of Some Medicinal Plants Against Human Pathogenic Bacteria.” Journal of Tropical Medicine 2019: 1895340. 31065287 10.1155/2019/1895340 PMC6466868

[fsn370912-bib-0050] Motilva, M.‐J. , A. Serra , and A. Macià . 2013. “Analysis of Food Polyphenols by Ultra High‐Performance Liquid Chromatography Coupled to Mass Spectrometry: An Overview.” Journal of Chromatography. A 1292: 66–82. 23369748 10.1016/j.chroma.2013.01.012

[fsn370912-bib-0051] Otitolaiye, C. , A. Omonkhua , K. Oriakhi , E. Okello , I. Onoagbe , and F. Okonofua . 2023. “Phytochemical Analysis and In‐Vitro Antioxidant Potential of Aqueous and Ethanol Extracts of Irvingia Gabonensis Stem Bark.” Pharmacognosy Research 1.

[fsn370912-bib-0052] Perna, A. , A. Simonetti , I. Intaglietta , A. Sofo , and E. Gambacorta . 2012. “Metal Content of Southern Italy Honey of Different Botanical Origins and Its Correlation With Polyphenol Content and Antioxidant Activity: Honey: Metal and Polyphenol Contents.” International Journal of Food Science and Technology 47, no. 9: 1909–1917. 10.1111/j.1365-2621.2012.03050.x.

[fsn370912-bib-0053] Rane, H. S. , S. M. Bernardo , S. M. Raines , J. L. Binder , K. J. Parra , and S. A. Lee . 2013. “ *Candida Albicans* VMA3 Is Necessary for V‐ATPase Assembly and Function and Contributes to Secretion and Filamentation.” Eukaryotic Cell 12, no. 10: 1369–1382. 23913543 10.1128/EC.00118-13 PMC3811332

[fsn370912-bib-0054] Rashmi, R. , S. B. Magesh , K. M. Ramkumar , S. Suryanarayanan , and M. V. SubbaRao . 2018. “Antioxidant Potential of Naringenin Helps to Protect Liver Tissue From Streptozotocin‐Induced Damage.” Reports of Biochemistry and Molecular Biology 7, no. 1: 76. 30324121 PMC6175592

[fsn370912-bib-0055] Said, Z. B.‐O. S. , Z. Bey‐Ould Si Said , H. Haddadi‐Guemghar , et al. 2016. “Essential Oils Composition, Antibacterial and Antioxidant Activities of Hydrodistillated Extract of *Eucalyptus Globulus* Fruits.” Industrial Crops and Products 89: 167–175.

[fsn370912-bib-0056] Sait, S. , S. Hamri‐Zeghichi , L. Boulekbache‐Makhlouf , et al. 2015. “HPLC‐UV/DAD and ESI‐MSn Analysis of Flavonoids and Antioxidant Activity of an Algerian Medicinal Plant: Paronychia Argentea Lam.” Journal of Pharmaceutical and Biomedical Analysis 111: 231–240. 25910047 10.1016/j.jpba.2015.03.027

[fsn370912-bib-0057] Sales, P. M. , P. M. Souza , L. A. Simeoni , P. O. Magalhães , and D. Silveira . 2012. “α‐Amylase Inhibitors: A Review of Raw Material and Isolated Compounds From Plant Source.” Journal of Pharmacy & Pharmaceutical Sciences 15, no. 1: 141–183. 22365095 10.18433/j35s3k

[fsn370912-bib-0058] Sharaf, M. , H. I. Hamouda , S. Shabana , et al. 2021. “Design of Lipid‐Based Nanocarrier for Drug Delivery Has a Double Therapy for Six Common Pathogens Eradication.” Colloids and Surfaces A: Physicochemical and Engineering Aspects 625: 126662. 10.1016/j.colsurfa.2021.126662.

[fsn370912-bib-0059] Tatlow, D. , S. Poothencheri , R. Bhangal , and C. Tatlow . 2015. “Novel Method for Rapid Reversal of Drug Toxicity: A Case Report.” Clinical and Experimental Pharmacology & Physiology 42, no. 4: 389–393. 25586596 10.1111/1440-1681.12358

[fsn370912-bib-0060] Toma, L. , G. M. Sanda , L. S. Niculescu , M. Deleanu , A. V. Sima , and C. S. Stancu . 2020. “Phenolic Compounds Exerting Lipid‐Regulatory, Anti‐Inflammatory and Epigenetic Effects as Complementary Treatments in Cardiovascular Diseases.” Biomolecules 10, no. 4: 641. 32326376 10.3390/biom10040641 PMC7226566

[fsn370912-bib-0061] Türkan, F. , M. N. Atalar , A. Aras , İ. Gülçin , and E. Bursal . 2020. “ICP‐MS and HPLC Analyses, Enzyme Inhibition and Antioxidant Potential of Achillea Schischkinii Sosn.” Bioorganic Chemistry 94: 103333. 31677859 10.1016/j.bioorg.2019.103333

[fsn370912-bib-0062] Van Acker, S. A. , M. N. Tromp , D. H. Griffioen , et al. 1996. “Structural Aspects of Antioxidant Activity of Flavonoids.” Free Radical Biology and Medicine 20, no. 3: 331–342. 8720903 10.1016/0891-5849(95)02047-0

[fsn370912-bib-0063] Vaou, N. , E. Stavropoulou , C. Voidarou , C. Tsigalou , and E. Bezirtzoglou . 2021. “Towards Advances in Medicinal Plant Antimicrobial Activity: A Review Study on Challenges and Future Perspectives.” Microorganisms 9, no. 10: 2041. 34683362 10.3390/microorganisms9102041 PMC8541629

[fsn370912-bib-0064] Veeraraghavan, V. P. , S. Hussain , J. P. Balakrishna , and S. K. Mohan . 2020. “Paronychia Argentea: A Critical Comprehensive Review on Its Diverse Medicinal Potential and Future as Therapeutics.” Pharmacognosy Journal 12, no. 5: 1172–1179.

[fsn370912-bib-0065] Wafa, B. A. , M. Makni , S. Ammar , et al. 2017. “Antimicrobial Effect of the Tunisian Nana Variety *Punica granatum* L. Extracts Against *Salmonella enterica* (Serovars Kentucky and Enteritidis) Isolated From Chicken Meat and Phenolic Composition of Its Peel Extract.” International Journal of Food Microbiology 241: 123–131. 27776287 10.1016/j.ijfoodmicro.2016.10.007

[fsn370912-bib-0066] Yakoubi, R. , S. Megateli , T. H. Sadok , and L. Gali . 2021. “Photoprotective, Antioxidant, Anticholinesterase Activities and Phenolic Contents of Different Algerian *Mentha Pulegium* Extracts.” Biocatalysis and Agricultural Biotechnology 34: 102038.

[fsn370912-bib-0067] Yuan, H. , Q. Ma , L. Ye , and G. Piao . 2016. “The Traditional Medicine and Modern Medicine From Natural Products.” Molecules 21, no. 5: 559. 27136524 10.3390/molecules21050559 PMC6273146

[fsn370912-bib-0068] Zahnit, W. , O. Smara , L. Bechki , et al. 2022. “Phytochemical Profiling, Mineral Elements, and Biological Activities of *Artemisia campestris* L. Grown in Algeria.” Horticulturae 8, no. 10: 914.

[fsn370912-bib-0069] Zahnit, W. , O. Smara , L. Bechki , M. Dekmouche , and C. Bensouici . 2023. “IN‐VITRO Assessment of Anti‐Cholinesterase, Anti‐Lipase, Antioxidant Activities and Photoprotective Effect of Algerian Fagonia Bruguieri DC Extracts.” Pharmaceutical Chemistry Journal 57, no. 1: 89–100.

[fsn370912-bib-0070] Zeinali, M. , S. A. Rezaee , and H. Hosseinzadeh . 2017. “An Overview on Immunoregulatory and Anti‐Inflammatory Properties of Chrysin and Flavonoids Substances.” Biomedicine & Pharmacotherapy 92: 998–1009. 28609844 10.1016/j.biopha.2017.06.003

[fsn370912-bib-0071] Zengin, G. , M. J. Rodrigues , H. H. Abdallah , et al. 2018. “Combination of Phenolic Profiles, Pharmacological Properties and In Silico Studies to Provide New Insights on Silene Salsuginea From Turkey.” Computational Biology and Chemistry 77: 178–186. 30336375 10.1016/j.compbiolchem.2018.10.005

[fsn370912-bib-0072] Zengin, G. , C. Sarikurkcu , A. Aktumsek , R. Ceylan , and O. Ceylan . 2014. “A Comprehensive Study on Phytochemical Characterization of Haplophyllum Myrtifolium Boiss. Endemic to Turkey and Its Inhibitory Potential Against Key Enzymes Involved in Alzheimer, Skin Diseases and Type II Diabetes.” Industrial Crops and Products 53: 244–251.

